# Low protein-induced increases in FGF21 drive UCP1-dependent metabolic but not thermoregulatory endpoints

**DOI:** 10.1038/s41598-017-07498-w

**Published:** 2017-08-15

**Authors:** Cristal M. Hill, Thomas Laeger, Diana C. Albarado, David H. McDougal, Hans-Rudolf Berthoud, Heike Münzberg, Christopher D. Morrison

**Affiliations:** 1 Pennington Biomedical Research Center, Baton Rouge, LA 70808 USA; 2 Department of Experimental Diabetology (DIAB), German Institute of Human Nutrition Potsdam-Rehbruecke (DIfE), Arthur-Scheunert-Allee 114-116, 14558 Nuthetal, Germany

## Abstract

Dietary protein restriction increases adipose tissue uncoupling protein 1 (UCP1), energy expenditure and food intake, and these effects require the metabolic hormone fibroblast growth factor 21 (FGF21). Here we test whether the induction of energy expenditure during protein restriction requires UCP1, promotes a resistance to cold stress, and is dependent on the concomitant hyperphagia. Wildtype, *Ucp1-*KO and *Fgf21-*KO mice were placed on control and low protein (LP) diets to assess changes in energy expenditure, food intake and other metabolic endpoints. Deletion of *Ucp1* blocked LP-induced increases in energy expenditure and food intake, and exacerbated LP-induced weight loss. While LP diet increased energy expenditure and *Ucp1* expression in an FGF21-dependent manner, neither LP diet nor the deletion of *Fgf21* influenced sensitivity to acute cold stress. Finally, LP-induced energy expenditure occurred even in the absence of hyperphagia. Increased energy expenditure is a primary metabolic effect of dietary protein restriction, and requires both UCP1 and FGF21 but is independent of changes in food intake. However, the FGF21-dependent increase in UCP1 and energy expenditure by LP has no effect on the ability to acutely respond to cold stress, suggesting that LP-induced increases in FGF21 impact metabolic but not thermogenic endpoints.

## Introduction

Over 40 years ago Rothwell, Stock and colleagues demonstrated that low protein (LP) diets increase energy expenditure (EE)^1, 2^. Subsequent work from the Stock lab demonstrated that LP diets increase *Ucp1* gene expression in both BAT and WAT^3^, implying that UCP1-dependent thermogenesis contributed to the increase in EE. Recently, our lab provided an endocrine mechanism to explain this protein dependent regulation of EE, as we demonstrated that LP diets require the hormone FGF21 to increase EE and *Ucp1* expression and to alter food intake and body weight gain^4, 5^. These data are consistent with evidence that pharmacological FGF21 treatment acts both in the brain and directly on adipose tissue to increase EE, stimulate sympathetic outflow, and upregulate thermogenic markers in BAT and WAT^6–10^. Thus FGF21-dependent activation of thermoregulatory signaling is a key mediator of the metabolic effects of a low protein diet.

While FGF21 clearly influences EE, the extent to which UCP1-dependent increases in EE are required for the metabolic action of FGF21 remains unclear. Some studies have suggested FGF21 does not require UCP1 to increase EE^11^, while in other studies the deletion of UCP1 attenuates the effect of FGF21^12, 13^. Considering that protein restriction leads to a chronic upregulation of EE and remodeling of adipose tissue^4^, it also seems possible that such effects might alter the metabolic response to acute cold stress, and several prior studies have suggested that FGF21 may be increased by cold stress^7, 14–18^. Finally, protein restriction is also well known to increase food intake in addition to EE^19, 20^. From an energy balance perspective, this pattern is unusual, as hormones of energy balance (leptin) typically promote opposing changes in food intake and EE. Thus it seems possible that the changes in food intake and EE are interrelated, with either the increase in food intake being an adaptive response to the elevated EE (protecting against weight loss), or alternatively the increase in EE being an adaptive response to the increase in food intake (protecting against weight gain).

Here we describe a series of studies designed to test the relationship between food intake, energy expenditure, UCP1, and FGF21 using a model of dietary protein restriction. In these studies, LP-induced increases in EE required both FGF21 and UCP1. However, despite increasing EE, FGF21 and UCP1, protein restriction had no effect on body temperature or the response to acute cold stress. Interestingly, LP-induced hyperphagia was also not required for the increase in EE during protein restriction. Taken together, these data support a model in which LP-induced FGF21 drives UCP1-dependent increases in energy expenditure to influence metabolic but not thermogenic endpoints.

## Results

### LP-induced increases in energy expenditure occur at thermoneutrality and require UCP1

**Figure 1:**
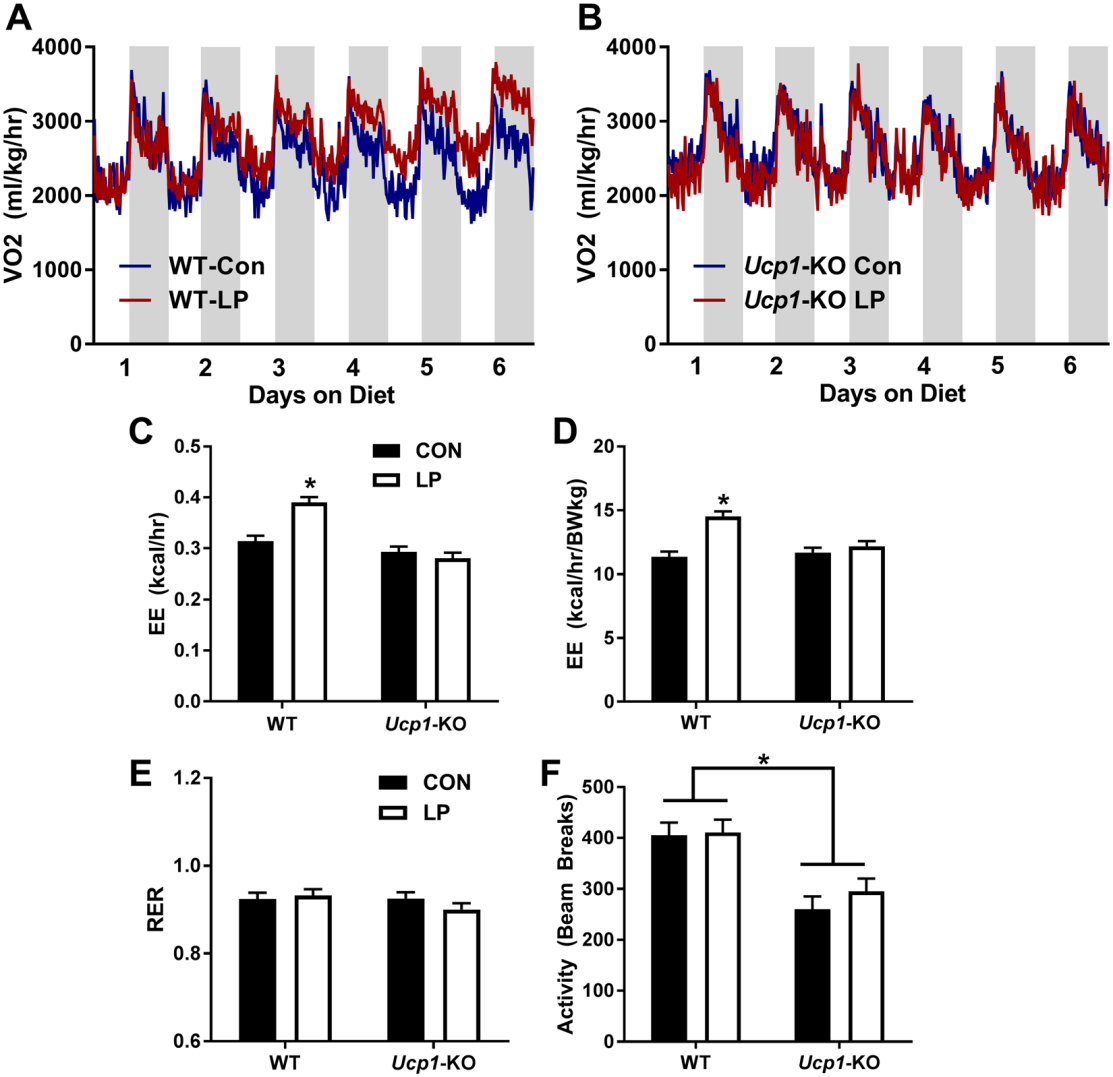
Low protein-induced increases in energy expenditure require UCP1. Wildtype and *Ucp1-*KO mice were placed on control or LP diet (8/group) at 28 °C, with energy expenditure being recorded for the first 6 days on diet. Raw oxygen consumption (VO2) following transition to control or LP diet in wildtype (**A**) and *Ucp1-*KO (**B**) mice. Average EE (**C**), EE normalized to body weight (**D**), RER (**E**), and activity (**F**) over days 5 and 6 of the experiment. *P < 0.05 LP vs. respective control.

Wildype and *Ucp1-*KO mice were placed on control and LP diets for 6 weeks at 28 °C, with energy expenditure measured over the first 7 days (Fig. 1A,B). Consistent with our previous studies, LP diet produced a significant increase in EE in wildtype mice (p < 0.05), which is evident regardless of whether the data are expressed on a per animal basis (Fig. 1C) or normalized to body weight (Fig. 1D). *Ucp1-*KO mice, however, exhibited no change in energy expenditure (Fig. 1B,C,D). LP diet did not affect respiratory exchange ratio (RER; Fig. 1E) in either WT or *Ucp1*-KO mice, and although *Ucp1-*KO mice were less active, there was no effect of diet on activity within either WT or *Ucp1-*KO mice (Fig. 1F). Thus UCP1 is required for LP-induced increases in whole body energy expenditure.

### Lack of UCP1 enhanced LP-induced weight loss in part by reversing the effect of LP on food intake

**Figure 2:**
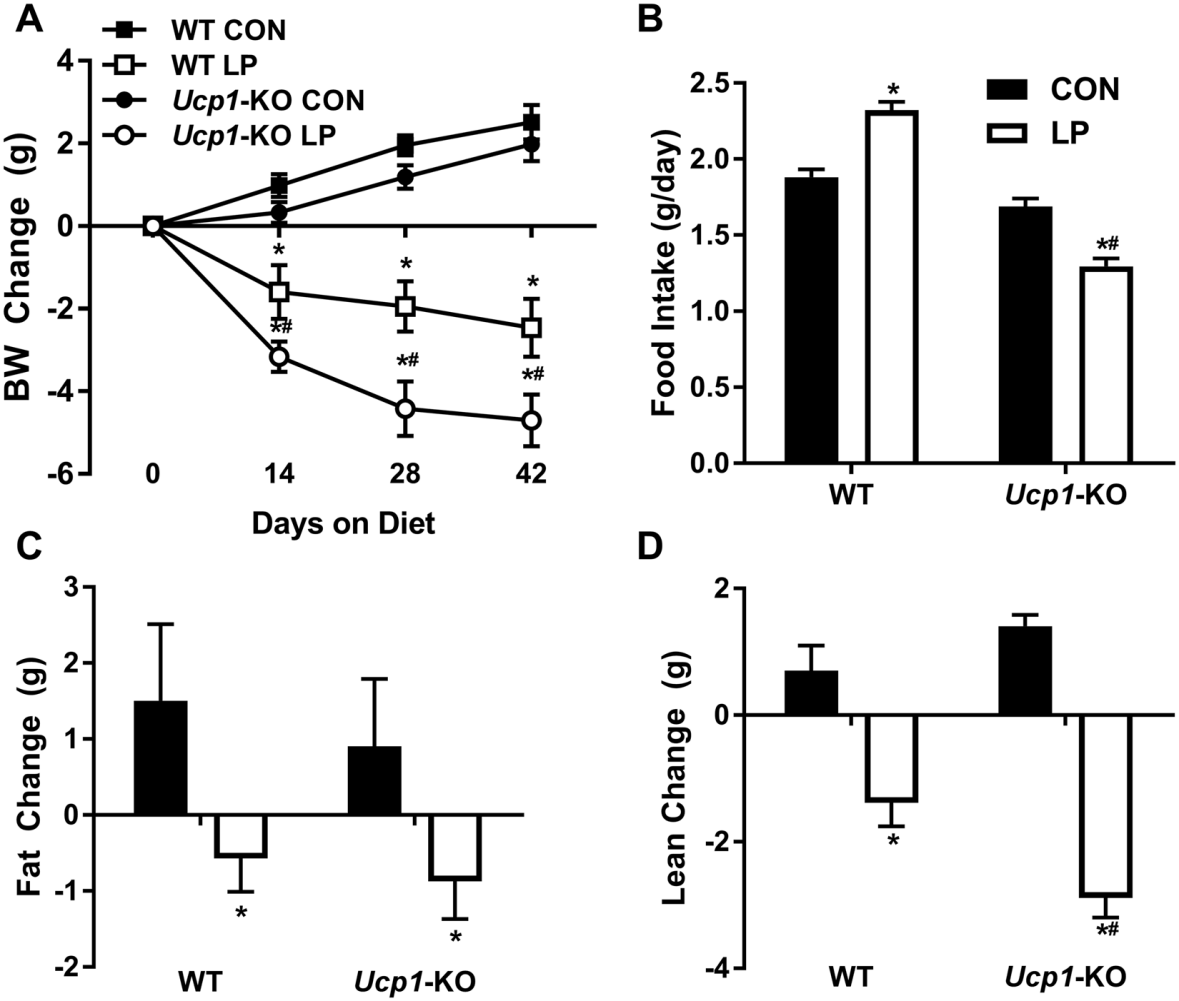
UCP1 deletion enhanced low protein-induced weight loss in part by reversing the effect of low protein on food intake. Body weight change (**A**), average daily food intake (**B**), fat (**C**) and lean mass (**D**) gain in wildtype and *Ucp1-*KO mice consuming control or LP diet (8/group) for 6 weeks at 28 °C. *P < 0.05 LP vs. respective control. ^#^P < 0.05 WT-LP vs. *Ucp1-*LP

After 7 days mice were removed from the metabolic chambers but remained on diet for 6 weeks in total. Consistent with our previous studies, the LP diet increased food intake (p < 0.01; Fig. 2B) but reduced body weight gain (p < 0.001; Fig. 2A) in WT mice, and these changes were associated with reductions in both fat (p < 0.001; Fig. 2C) and lean (p < 0.001; Fig. 2D) gain. *Ucp1-*KO mice failed to exhibit hyperphagia, and in fact reduced food intake when on LP diet (p < 0.01; Fig. 2B). As a result, *Ucp1-*KO mice lost significantly more weight on LP than WT mice (p < 0.001; Fig. 2A), even though they did not increase EE. In summary, deletion of UCP1 exacerbates LP-induced weight loss and reveals an anorectic effect of LP diet.

### Effect of LP on FGF21, liver and BAT metabolic genes in Ucp1-KO mice

**Figure 3:**
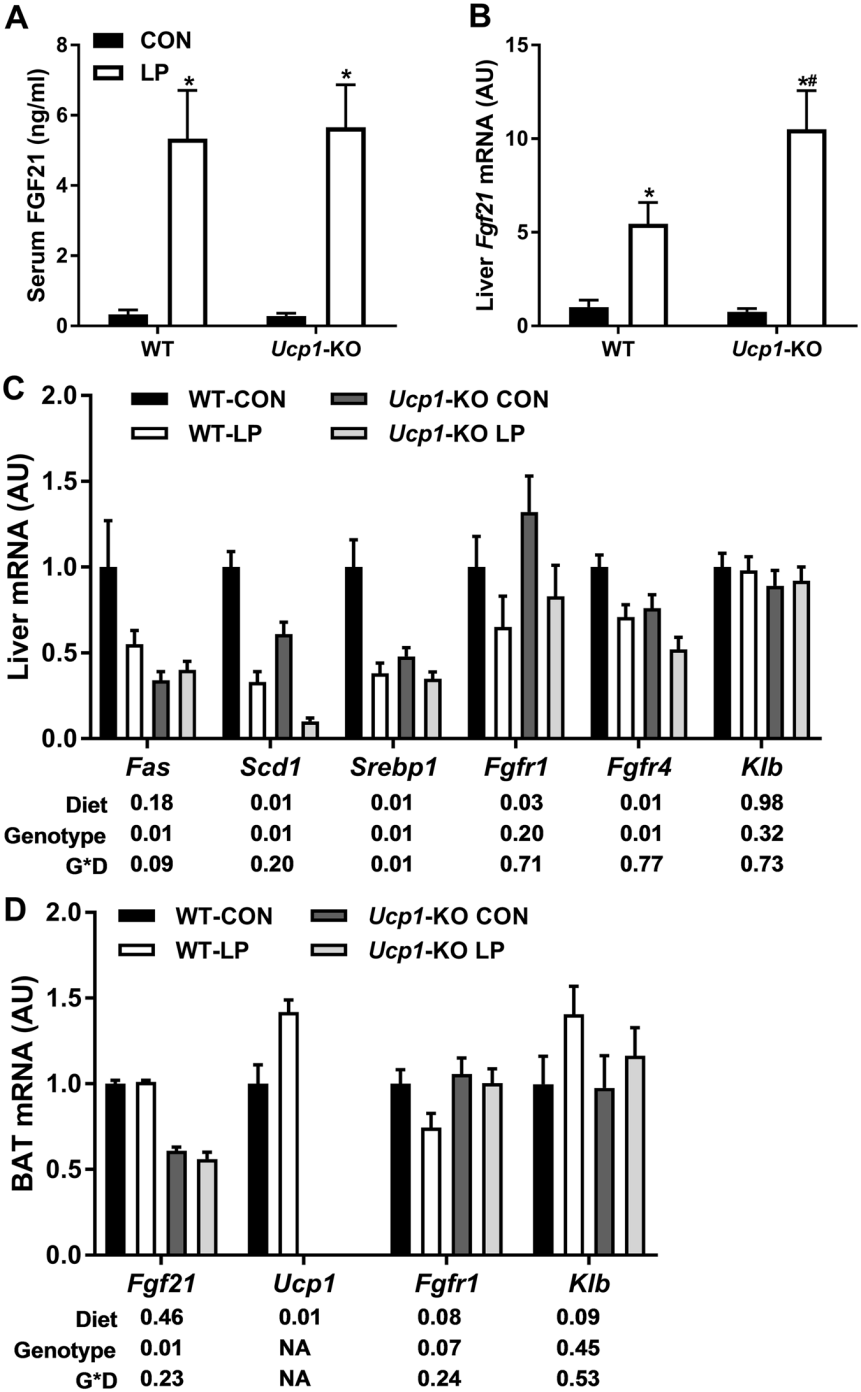
Effect of low protein on FGF21 and liver and BAT metabolic gene expression in *Ucp1-*KO mice. Tissues were collected from wildtype and *Ucp1-*KO mice after consuming control or LP diet (8/group) for 6 weeks. Serum FGF21 was measured via ELISA (**A**), while liver *Fgf21* (**B**,**C**) and BAT (**D**) gene expression was measured via real-time PCR. *P < 0.05 LP vs respective control; ^**+**^P < 0.05 WT-CON vs. *Ucp1-*CON; ^#^P < 0.05 WT-LP vs. *Ucp1-*LP. Probability values for the two-way ANOVA main effects of diet and genotype, and their interaction (G*D) are provided below the respective mRNA in **C** and **D**.

After 6 weeks on diet mice were sacrificed to assess key metabolic endpoints. As expected, LP produced a robust increase in circulating FGF21 protein (p < 0.01; Fig. 3A) and liver *Fgf21* mRNA expression (p < 0.01; Fig. 3B), and this effect was intact in *Ucp1-*KO mice. Consistent with our previous work, LP diet also reduced the expression of genes associated with lipogenesis within the liver (*Fas*, *Scd1* and *Srebp1*;Fig. 3C). *Ucp1-*KO mice exhibited lower levels of all these genes at baseline (p < 0.01), and LP diet reduced the expression of *Scd1* but not *Fas* or *Srebp1* in *Ucp1-*KO mice. We also measured expression of the FGF21 receptor proteins *Fgfr1*, *Fgfr4* and beta-Klotho (*Klb*) within the liver (Fig. 1C). LP diet reduced the expression of *Fgfr1* and *Fgfr4* across both genotypes (p < 0.03), but had no effect on *Klb*. Within BAT, LP diet did not alter *Fgf21* mRNA expression in either WT or *Ucp1-*KO mice (Fig. 3D), although *Ucp1-*KO mice had lower baseline levels of *Fgf21* mRNA. Consistent with the increase in EE, LP diet significantly increased BAT *Ucp1* mRNA expression in WT mice (p < 0.001; Fig. 3D), while *Ucp1* mRNA expression was undetectable in *Ucp1-*KO mice. Finally, we observed no clear pattern for changes in *Fgfr1* or *Klb* within BAT, and *Fgfr4* expression was below the limit of detection. These data demonstrate that LP-induced FGF21 is intact in *Ucp1-*KO mice, and therefore that their altered metabolic response to LP is due to an inability of FGF21 to engage UCP1-dependent mechanisms.

### LP-induced FGF21 increases energy expenditure but does not alter the response to acute cold stress

**Figure 4:**
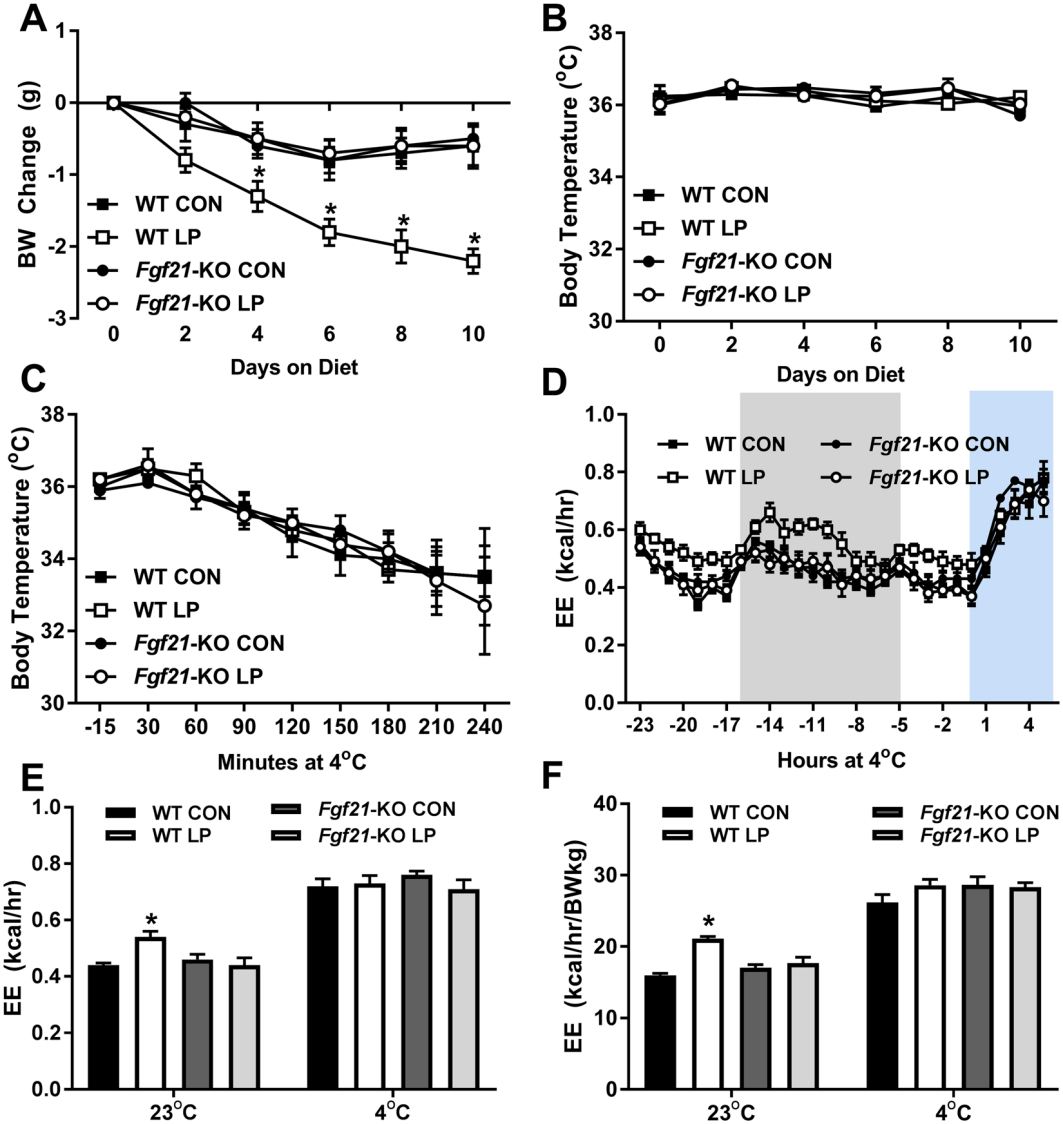
Low protein-induced FGF21 increases EE but does not alter the response to acute cold stress. WT and *Fgf21-*KO mice were placed on LP diet for 10 days (10/group) at room temperature, with body weight change (**A**) and rectal temperature (**B**) measured every two days. On day 10 food was removed and temperature was reduced to 4 °C for 6hrs, with rectal temperature (**C**) and EE (**D, E, F**) measured. Raw EE (**E**) and EE normalized to body weight (**F**) for the 24 hr prior to cold exposure (23 °C) and the final 3 hr of cold exposure (4 °C). Gray shading in Panel D represents lights off, and the blue shading represents the period of cold exposure. *P < 0.05 LP vs. respective control.

Dietary protein restriction induces persistent increases in energy expenditure and upregulates thermogenic gene expression in both BAT and WAT^4^, suggesting that exposure to LP diet may enhance the metabolic response to acute cold stress. In addition, FGF21 has been separately linked to both the response to protein restriction and the response to cold stress, and thus loss of FGF21 should attenuate any beneficial effect of LP diet. To test this question, wildtype and *Fgf21-*KO mice were placed on control or LP diet for 10 days at room temperature (to increase EE in the WT mice) and were then subjected to an acute cold stress (6hr at 4 °C without food). Consistent with our previous work, LP diet led to a significant reduction in body weight in the WT mice (p < 0.001; Fig. 4A **)**, but had no effect on *Fgf21-*KO mice. During these 10 days of diet exposure, there was no change in rectal temperature (Fig. 4B), but a significant increase in EE was observed in WT but not *Fgf21*-KO mice consuming LP mice on Days 9–10 (p < 0.01; Fig. 4D,E and F). Despite their increased energy expenditure, WT-LP mice responded similarly to the acute cold, with rectal temperature dropping similarly in all animals (Fig. 4C). Cold exposure also led to an increase in EE in all animals that obscured any effect of LP in WT mice (Fig. 4D,E and F). These data suggest that even though LP increases basal EE it has no effect on the metabolic response to acute cold stress, and that FGF21 is required for LP-induced increases in EE but not acute cold-induced increases in EE.

### Effect of low protein and FGF21 deletion on metabolic endpoints following acute cold stress

**Figure 5:**
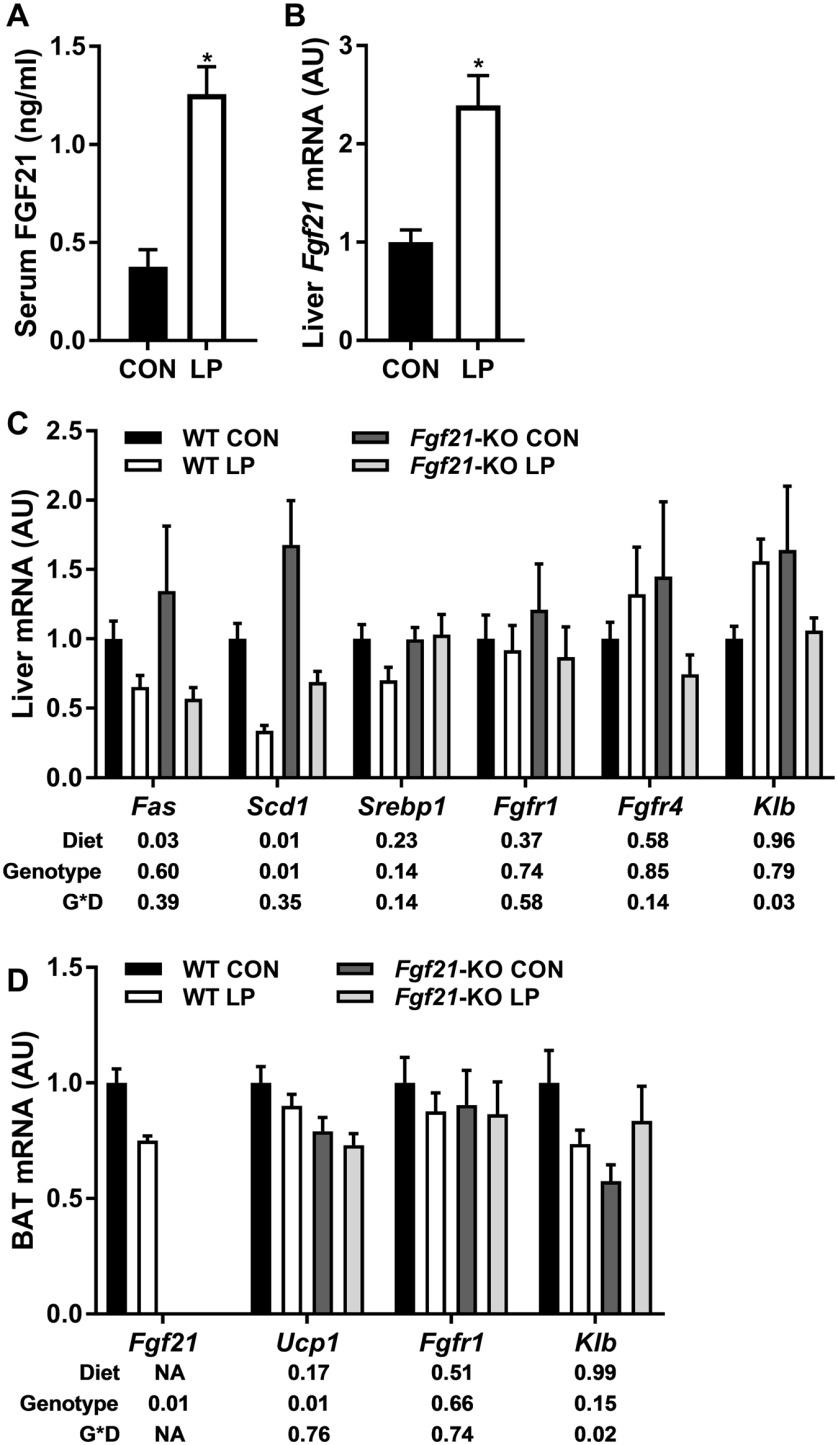
Effect of low protein diet and FGF21 deletion on metabolic endpoints following acute cold stress. WT and *Fgf21*-KO mice were placed on LP for 10 days, and on day 10 food was removed and temperature was reduced to 4 °C. Mice were sacrificed after 6hrs at 4 °C, and serum FGF21 (**A**), liver *Fgf21* mRNA (**B**), liver (**C**) and BAT (**D**) mRNA expression was measured. 10/group. *P < 0.05 LP vs. respective control. Probability values for the two-way ANOVA main effects of diet and genotype, and their interaction (G*D) are provided below the respective mRNA in **C** and **D**.

Mice were sacrificed at the end of the 6hr cold stress to assess changes in FGF21 or metabolic markers in liver and BAT. As above, LP diet increased both circulating FGF21 protein levels (p < 0.001; Fig. 5A) and liver *Fgf21* mRNA expression (p < 0.001; Fig. 5B) in WT mice (FGF21 is not detected in *Fgf21*-KO mice). However, the increment of this LP-induced FGF21 following cold exposure was smaller than in previous studies conducted at room temperature or thermoneutrality (Fig. 3). LP diet also reduced hepatic expression of *Fas* and *Scd1* in both WT and *Fgf21-*KO mice (Fig. 5C), but produced no consistent effects on *Klb*, *Fgfr1* or *Fgfr4*. Interestingly, LP diet decreased *Fgf21* mRNA expression within BAT after cold exposure (p < 0.01; Fig. 5D), and there was no effect of LP to alter BAT *Ucp1, Klb*, or *Fgfr1* after cold exposure (Fig. 5E).

### Hyperphagia is not necessary for LP-induced increases in energy expenditure

**Figure 6:**
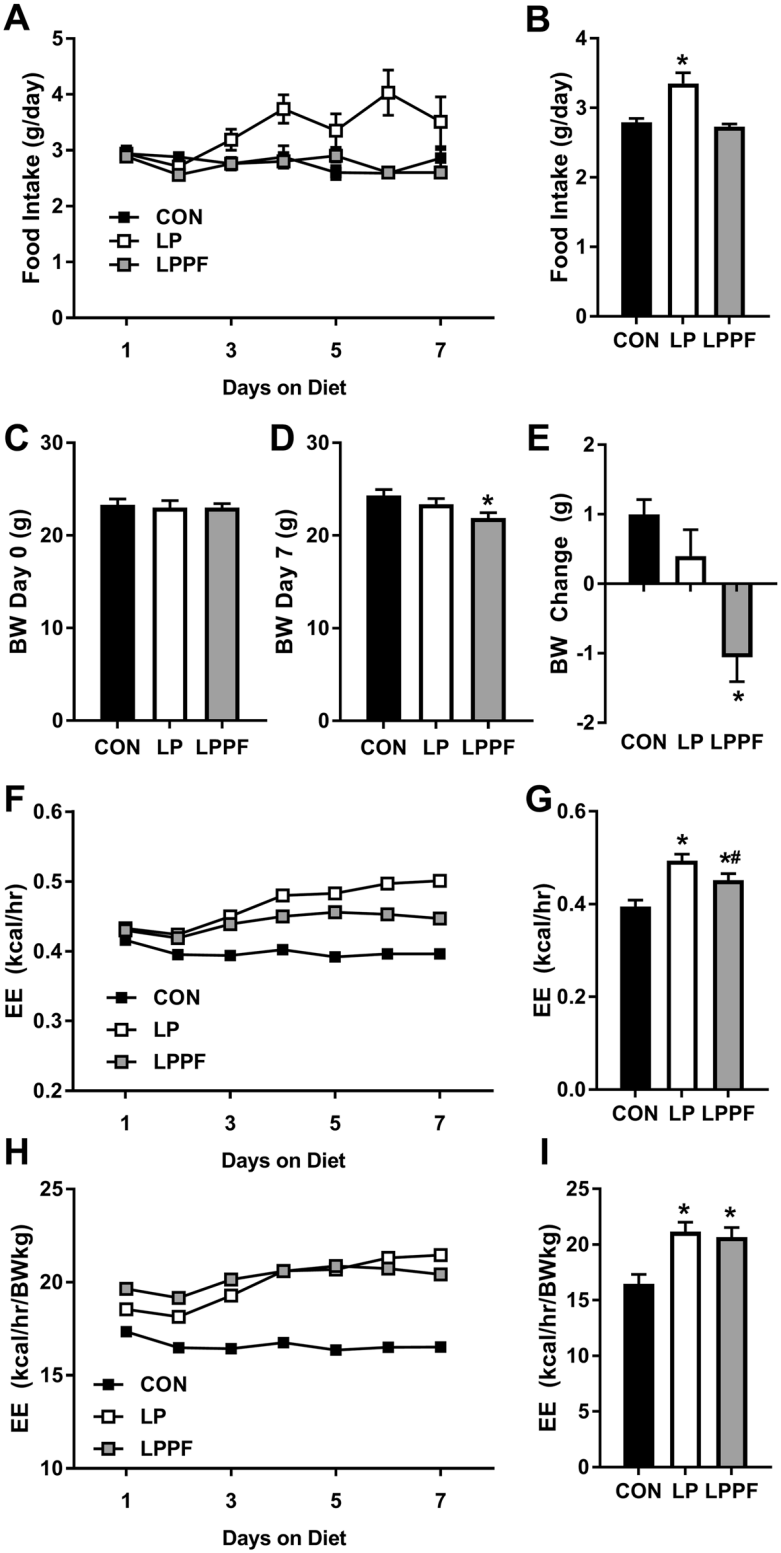
Hyperphagia is not necessary for low protein-induced increases in energy expenditure. Wildtype mice were placed on control or LP diet ad libitum for 7 days, or were placed on LP but pair-fed to the daily intake of the control group (LPPF) to prevent hyperphagia. Daily food intake (**A**,**B**), body weight on day 0 (**C**) and day 7 (**D**), and BW change for the 7 days (**E**). Unadjusted average daily energy expenditure (**F**) and averaged across days 5–7 (**G**) and EE adjusted for body weight on day 7 (**H** and **I**). 8 mice/group. *P < 0.05 vs. control. ^#^P < 0.05 vs. LP.

Dietary protein restriction is known to increase both food intake and EE, but the relationship between these two endpoints is unclear. One interpretation of the above data is that increased EE is the primary outcome of dietary protein restriction, and that changes in body weight, food intake and metabolic gene expression are secondary to this effect. If increased EE is indeed independent of the hyperphagia, then preventing LP-induced hyperphagia should have no effect on the induction of energy expenditure. To test this question, wildtype mice were placed on control or LP diet ad libitum for 7 days, or a third group was placed on LP but pair-fed to the daily food intake of the controls (LPPF) for 7 days, thereby preventing LP-induced hyperphagia. Consistent with previous work, the LP diet significantly increased food intake (p = 0.004; Fig. 6A,B), and pair-feeding successfully prevented this increase. Although LP reduced BW gain, this effect did not reach statistical significance (p = 0.24; Fig. 6E). However, pair-feeding exacerbated the effect of LP, such that LPPF mice lost more weight than LP mice (p = 0.005; Fig. 6E). As previously shown, exposure to the LP diet led to a significant increase in EE, which was apparent regardless of whether the data was expressed per mouse (p < 0.001; Fig. 6F and G) or normalized to BW on Day 7 (p < 0.001; Fig. 6H and I). On a per mouse basis, EE also increased in LPPF mice (p = 0.008; Fig. 6G), although the effect was reduced relative to mice on LP ad libitum (p = 0.05). However, normalization for body weight on Day 7 removes this difference between LP and LPPF (Fig. 6I), suggesting that the difference in body weight explains the difference in EE between LP and LPPF animals. Taken together, these data suggest that LP-induced increases in energy expenditure occur independently of the increase in food intake, and indeed that the increase in food intake serves to attenuate weight loss induced by elevated EE.

## Discussion

It is well known that restriction of dietary protein intake, without energy restriction, is sufficient to increase both food intake and energy expenditure^1, 2, 19, 21–24^. Recent work from our lab and others indicates that the endocrine hormone FGF21 is increased by protein restriction and required for these changes^4, 5, 25–29^. However, several additional questions remain regarding the mechanism through which FGF21 acts in the context of protein restriction, and whether these FGF21-dependent effects on food intake, EE and body weight are induced independently from one another or are instead interconnected.

UCP1-dependent thermogenesis is a key driver of energy expenditure in rodents. We have previously demonstrated that protein restriction increases UCP1 and promotes the browning of WAT, and that these effects require FGF21^4^. This outcome is consistent with evidence that pharmacological FGF21 treatment increases EE, UCP1, and promotes WAT browning^6–8, 10, 30, 31^. However, the extent to which UCP1 is required for pharmacological responses to FGF21 remains controversial, as some studies suggest that FGF21 does not require UCP1 to increase EE^11^, while in other studies the deletion of UCP1 attenuates the effect of FGF21^12, 13, 32^. We contend that mediating adaptive responses to protein restriction is a key physiological role for FGF21, and therefore studying the UCP1-FGF21 interaction in this context provides unique physiological insight. As such, we first tested whether metabolic responses to a low protein (LP) diet require UCP1. As expected, the LP diet led to a series of metabolic changes in wildtype mice, most notably an increase in EE, increase in food intake, and an inhibition of body weight and fat gain. Contrastingly, EE was unchanged in *Ucp1-*KO mice on the LP diet, despite a normal increase of circulating FGF21. This outcome suggests that the recruitment of UCP1 is a key mechanism through which dietary protein restriction increases EE, and that other metabolic systems cannot compensate for the absence of UCP1. While LP did not increase EE in *Ucp1-*KO mice, it did produce unique changes in BW gain and food intake. Perhaps the most striking observation was that LP diet increased food intake in the WT mice but decreased food intake in *Ucp1-*KO mice, and as a result *Ucp1-*KO mice lost more weight on LP than WT mice. This response is reminiscent of the work of Samms *et al*., where FGF21 treatment increased food intake in WT mice but decreased food intake in *Ucp1-*KO mice^12^, and of Wanders *et al*., where food intake increased in WT but decreased in *Ucp1-*KO mice eating a methionine restricted diet^13^. Together, these studies suggest that UCP1 deletion uncovers an anorectic effect of FGF21. While the mechanism underlying this divergent feeding response is unknown, one interpretation is that the LP-induced increase in food intake in wildtype animals represents an adaptive response to the increase in energy expenditure. This conclusion is also supported by the pair-feeding study, where preventing LP-induced hyperphagia actually enhanced weight loss. Finally, *Ucp1*-KO mice are known to exhibit unique physiological changes when housed at temperatures below thermoneutrality^33^, and therefore this experiment was conducted entirely at thermoneutrality (28 °C). Indeed, cold exposure triggers increases in adipose tissue *Fgf21* mRNA expression in *Ucp1-*KO mice that are not observed in wildtype mice^34^. The observation that the LP-induced increases in EE that we previously observed at 23 °C are replicated at 28 °C indicates that this effect represents a specific engagement of adaptive thermogenesis. Interestingly, our work contradicts a recent study by Maida and colleagues^25^ which suggests that UCP1 was not required for the metabolic effects of a low protein diet. While EE was not measured directly, Maida *et al*. observed a significant increase in body temperature and reduction in feed efficiency in both WT and *Ucp1-*KO mice on low protein. However, there was consistency with regard to effects on food intake and body weight, as both studies detected a reduction in food intake and BW in *Ucp1-*KO mice fed low protein diet.

Since dietary protein restriction browns white fat and increases energy expenditure and *Ucp1* mRNA expression, we next hypothesized that mice previously exposed to a LP diet might exhibit a more robust response to acute cold stress. We also tested whether FGF21 is required this effect, since LP-induced effects require FGF21^4, 5^ and FGF21 may be increased by cold stress^7, 14–18, 34^. Importantly, it should be noted that shivering-induced thermogenesis (BAT independent) plays a key role in the maintenance of body temperature in response to an acute cold challenge. When exposed to acute cold, animals initially shiver to maintain core body temperature until BAT thermogenesis is mobilized. Because mice on LP diet already exhibit increased BAT UCP1, browned iWAT and increased baseline EE, we predicted that the LP group would more readily and rapidly engage BAT thermogenesis in response to cold. Thus, we predicted three outcomes: (1) Prior exposure to dietary protein restriction would allow mice to more readily maintain temperature during acute cold stress, (2) cold-induced increases in EE would be lower in LP mice, because BAT thermogenesis is more energy efficient than shivering thermogenesis, and (3) these LP-induced effects would be absent in *Fgf21-*KO mice. As expected, the LP diet produced a significant increase in systemic FGF21 levels, although in this study the magnitude of the increase was reduced compared to our earlier experiments. Nevertheless, LP diet reduced body weight and increased EE in wildtype mice, and both of these effects were lost in *Fgf21-*KO mice. However, when mice were exposed to 6hrs of cold exposure (without food), body temperature decreased similarly and EE increased similarly in all groups. This outcome suggests that prior exposure to LP had no effect on the ability to respond to acute cold. In addition, even though *Fgf21-*KO mice failed to increase EE in response to LP diet, they were fully capable of increasing EE when exposed to acute cold stress. Finally, the substantial increase in EE produced by the cold stress obscured the more modest effect of LP to increase EE that was observed at both 23 °C and 28 °C. The observation that LP increases EE but not body temperature or cold tolerance suggests that LP animals more readily dissipate heat, which is consistent with prior work indicating that protein restriction increases surface body temperature^35^ but contrasts a recent study observing an increased core body temperature^25^. Taken together, these data demonstrate that FGF21 is required for LP but not cold-induced increases in EE. In addition, even though prior exposure to the LP diet increases FGF21 and UCP1, browns iWAT, and increases EE^4^, these responses appear to be largely irrelevant to the thermogenic response to acute cold exposure.

Prior work provides strong evidence that FGF21 can increase energy expenditure and reduce body weight, but its effects on food intake are less clear. Multiple studies suggest that FGF21 treatment either has no effect or increases food intake^8, 10, 12, 36^, while other studies suggest FGF21 or FGF21 analogs act to reduce food intake^37–39^ or to selectively inhibit sweet and alcohol consumption^40, 41^. In settings of dietary protein restriction, we and others have observed a persistent increase in both food intake and EE^1, 2, 19, 21–24, 42^, and these effects require FGF21^4, 5^. Nevertheless, the causal relationship between the two endpoints is less clear. Does the increase in EE drive a compensatory increase in food intake, or instead does the increase in food intake drive a compensatory increase in EE? Effectively testing this question requires that one variable be held constant while testing if LP still influences the other. As described above, the deletion of *Ucp1* prevented LP-induced increases in EE, effectively clamping EE. Interestingly, in this context LP diet did not increase food intake but in fact decreased it. Thus one interpretation of this experiment is that increased EE is required for increased food intake, although the deletion of *Ucp1* likely produces a number of metabolic adaptations that make interpretation problematic. To test the opposite comparison wildtype mice were placed on the LP diet but were pair-fed to the food intake of the control, thereby preventing LP-induced hyperphagia. Importantly, the LP-induced increase in EE is fully intact in this setting, demonstrating that LP-induced increases in EE do not require an increase in food intake. These two experiments together suggest that increased EE is a primary metabolic effect of LP diet, and that increases in total food intake may be a compensatory response to resist the resulting weight loss. This interpretation is consistent with the fact that pair-fed mice lost more weight than LP mice, and with older work suggesting that low protein diets activate hypothalamic NPY/AgRP neurons^42, 43^.

Taken together, these data lead to four notable conclusions. (1) Consistent with our previous work, LP-diet increases FGF21 and FGF21 is required for metabolic responses to LP diet. (2) LP-induced increases in EE require UCP1, and loss of UCP1 reverses the feeding effect of LP diet, (3) Despite the FGF21-dependent increase in EE and recruitment of UCP1, neither LP diet nor FGF21 deletion influence the adaptive response to acute cold stress, and finally (4) the induction of EE seems to be a primary adaptive response to protein restriction, while increases in total food intake seem to be secondary to the increase in EE. As such, these data support a model in which LP-induced FGF21 drives UCP1-dependent increases energy expenditure to influence metabolic but not thermogenic endpoints.

## Materials and Methods

### Animals and Diets

All procedures involving animals were approved by the PBRC Institutional Animal Care and Use Committee, and were performed in accordance with the guidelines and regulations of the NIH Office of Laboratory Animal Welfare. Male C57BL6 mice (Jackson Lab) were used in all studies. *Ucp1-*deficient mice on the B6 background were derived from an established colony at PBRC^13, 44^, while *Fgf21-*deficient mice on the B6 background were kindly provided by Dr. Steven Kliewer (UT Southwestern) as described previously^45^. Control and LP diets were formulated and produced by Research Diets as previously described^4, 5^, and were designed to be isocaloric by equally varying protein and carbohydrate while keeping fat constant. Control diet contained 20% casein (by weight) as the protein source, while the LP diet contained 5% casein. Animals were single housed in 12:12hr light:dark cycle with ad libitum access to food or water unless otherwise noted. For all experiments, single housed mice were transferred from chow to the control diet for approximately 5 days, at which point a random subgroup of animals were transferred to the respective LP diet. At the end of the study mice were sacrificed during the mid-light cycle in the fed state (unless otherwise noted) using acute exposure to CO_2_ followed by rapid decapitation. Trunk blood was also collected at sacrifice, allowed to clot overnight at 4 °C, centrifuged at 3000xg and serum collected. Tissues were collected and snap frozen in liquid nitrogen for further analysis.

### Experimental Design

#### Experiment 1: Effect of protein restriction on Ucp1-deficient mice housed at thermoneutrality

Wildtype and *Ucp1-*KO male mice (~3 months of age) were placed on control or LP diet for 6 weeks (8 mice/diet/genotype), and were housed at 28 °C for the entire study period. Initial transition to diet treatments occurred within metabolic chambers (Oxymax, Columbus Instruments), with animals moved to standard housing after 1 week. Mice remained on their respective diets for 6 weeks, with body weight and food intake measured weekly. Body composition was measured via TD-NMR (Bruker Minispec) at the start of experimental diets (Day 0), the final day of OxyMax metabolic analysis (Day 7) and on the day of sacrifice (Day 42).

#### Experiment 2: Effect of protein restriction and cold stress in Fgf21-deficient mice

Wildtype and *Fgf21-*KO male mice were entered into the study at approximately 3 months of age and were housed at room temperature (23 °C), placed on control or LP diet for 10 days (10 mice/diet/genotype), and food intake, body weight and rectal temperature recorded every 2 days. On day 9, half the mice in each group were placed into metabolic chambers to record baseline EE at 23 °C. On day 10, housing temperature for all mice was reduced to 4 °C for 6 hrs beginning at 11AM, with EE being recorded for the mice in metabolic chambers and rectal temperatures recorded every 30 minutes for the remaining mice. At 6hrs mice were sacrificed and tissues collected as described above.

#### Experiment 3: Effect of pair-feeding on LP-induced increases in energy expenditure in wildtype mice

At 3 months of age, male wildtype mice were randomly divided into three treatment groups and fed control or LP diet *ad libitum*, or were fed the LP diet but pair-fed to the daily intake of the control group (to prevent LP-induced hyperphagia). Mice were fed these diets for 7 days, with the entire experiment occurring within metabolic chambers (23 °C) to assess changes in energy expenditure. Food intake was measured daily, with body weight and composition measured at the beginning (Day 0) and end (Day 7) of the experiment.

#### Immunoassay Determination of FGF21

Concentrations of FGF21 in serum were determined in mice with an ELISA according to the procedure recommended by the manufacturer (no. RD291108200R, Mouse and Rat FGF-21 ELISA, BioVendor). The minimal detectable concentration of FGF21 with this assay was 18.4 pg/ml. For determination of serum FGF21, 50 µl of serum were diluted in 200 μl of dilution buffer before analysis.

#### Real-time PCR

RNA extraction and real-time PCR was conducted as described previously^42^. Total RNA was extracted from liver, iWAT, and BAT using TRIzol reagent following the manufacturer’s protocol (Invitrogen), with the addition of an RNeasy Lipid Tissue Mini Kit (Qiagen) for the BAT & iWAT. RNA purity and quantity was determined by spectrophotometry using a NanoDrop (Thermo Scientific). cDNA synthesis was performed with iScript (BioRad) and mRNA was quantified on the ABI 7900 platform using the ABI SYBR Green PCR Master Mix in optical 384-well plates (Applied Biosystems). Primer pairs were designed using the IDT RealTime qPCR Primer Design tool with at least one primer spanning an exon-exon boundary. Target gene expression was normalized with cyclophilin as the endogenous control. Primers used are as follows, written 5′ to 3′:

*Fas* For GGGATCTGGTGAAAGCTGTAG; Rev GTGTTCTCGTTCCAGGATCTG

*Scd-1* For CTGTACGGGATCATACTGGTTC; Rev CGTGCCTTGTAAGTTCTGTG

*Srebp1* For AGATTGTGGAGCTCAAAGACC; Rev CACTTCGTAGGGTCAGGTTC

*Klb* For CAGGGATATCTACATCACAGCC; Rev GTAGCCTTTGATTTTGACCTTGTC

*Fgf21* For CAAATCCTGGGTGTCAAAGC; Rev CATGGGCTTCAGACTGGTAC

*Fgfr1* For AAAGATCTGGTATCCTGTGCC; Rev TCGAGCTAAGCCAAAGTCTG

*Fgfr4* For TGTCAAATTCCGCTGTCCAG; Rev ACCACACTTTCCATCACCAG

*Cyc* For CTTCGAGC TGTTTGCAGACAAAGT; Rev AGATGCCAGGACCTGTATGCT.

### Statistical Analysis

Data were analyzed using the SAS software package (SAS V9, SAS Institute) using one-way, two-way, or repeated measures ANOVA using the general linear model procedure. When experiment-wide tests were significant, post-hoc comparisons were made using the LSMEANS statement with the PDIFF option, and represent least significant differences tests for pre-planned comparisons. All data are expressed as mean ± SEM, with a probability value of 0.05 considered statistically significant.

### Data Availability

The datasets generated and/or analyzed during the current study are available from the corresponding author on reasonable request.
